# Factors Influencing Resident Responsive Behaviors Toward Staff in Nursing Homes: A Systematic Review

**DOI:** 10.1093/geroni/igab046.1442

**Published:** 2021-12-17

**Authors:** Lori Weeks, Abubakar Mohamed Nassur, Fajr Haq, Viraji Rupasinghe, Carole Estabrooks, Yuting Song

**Affiliations:** 1 Dalhousie University, Dalhousie University, Nova Scotia, Canada; 2 University of Alberta, University of Alberta, Alberta, Canada; 3 University of Calgary, Calgary, Alberta, Canada; 4 Dalhousie University, Halifax, Nova Scotia, Canada; 5 University of Alberta, Edmonton, Alberta, Canada

## Abstract

When staff experience various types of resident responsive behaviors, this can lead to decreased quality of work-life and lower quality of care. We synthesized empirical quantitative and qualitative evidence on factors associated with resident responsive behaviors directed towards staff in nursing homes. We searched 12 bibliographic databases and "grey" literature with two key words: long-term care and responsive behaviors resulting in 7671 sources. Pairs of reviewers independently completed screening, data extraction, and risk of bias assessment. Based on extracted data, we developed a coding scheme of factors utilizing the ecological model as an organizational structure. We then applied the coding scheme to quantitative and qualitative articles and prepared narrative summaries for each factor. From 86 included studies (57 quantitative, 28 qualitative, 1 mixed methods), multiple factors emerged, such as staff training about responsive behaviors (individual level); staff approaches to care (interpersonal level); leadership, staffing resources, and physical environment (institutional level); and racism and patriarchy (societal level). Quantitative and qualitative results each provided key insights, such as qualitative results pertaining to leadership responses to reports of responsive behaviors, and quantitative findings on the impact of staff approaches to care on responsive behaviors. By synthesizing both quantitative and qualitative evidence, this review provides a comprehensive overview of factors associated with resident responsive behaviors towards staff. Our findings offer insights into promising factors for long-term care system and nursing home managers to address to strive to reduce responsive behaviors of residents toward staff in nursing homes.

